# An antipathogenic compound that targets the OxyR peroxide sensor in *Pseudomonas aeruginosa*

**DOI:** 10.1099/jmm.0.001341

**Published:** 2021-04-08

**Authors:** Hyo-Young Oh, Shivakumar S. Jalde, In-Young Chung, Yeon-Ji Yoo, Hye-Jeong Jang, Hyun-Kyung Choi, You-Hee Cho

**Affiliations:** ^1^​ Department of Pharmacy, College of Pharmacy and Institute of Pharmaceutical Sciences, CHA University, Gyeonggi-do 13488, Republic of Korea; ^2^​ Department of Medicinal Chemistry, Jungwon University, Goesan 28024, Republic of Korea

**Keywords:** antipathogenic, *Pseudomonas aeruginosa*, OxyR, oxidative stress, virulence

## Abstract

**Introduction:**

Antipathogenic or antivirulence strategy is to target a virulence pathway that is dispensable for growth, in the hope to mitigate the selection for drug resistance.

**Hypothesis/Gap Statment:**

Peroxide stress responses are one of the conserved virulence pathways in bacterial pathogens and thus good targets for antipathogenic strategy.

**Aim:**

This study aims to identify a new chemical compound that targets OxyR, the peroxide sensor required for the full virulence of the opportunistic human pathogen, *Pseudomonas aeruginosa *.

**Methodology:**

Computer-based virtual screening under consideration of the ‘eNTRy’ rules and molecular docking were conducted on the reduced form of the OxyR regulatory domain (RD). Selected hits were validated by their ability to phenocopy the *oxyR* null mutant and modulate the redox cycle of OxyR.

**Results:**

We first isolated three robust chemical hits that inhibit OxyR without affecting prototrophic growth or viability. One (compound 1) of those affected the redox cycle of OxyR in response to H_2_O_2_ treatment, in a way to impair its function. Compound 1 displayed selective antibacterial efficacy against *P. aeruginosa* in *Drosophila* infection model, without antibacterial activity against *Staphylococcus aureus *.

**Conclusion:**

These results suggest that compound 1 could be an antipathogenic hit inhibiting the *P. aeruginosa* OxyR. More importantly, our study provides an insight into the computer-based discovery of new-paradigm selective antibacterials to treat Gram-negative bacterial infections presumably with few concerns of drug resistance.

## Introduction

The rapid evolution and dissemination of antimicrobial resistance (AMR) represents a major threat to the global public health. Considering the complicated polymicrobial nature of the infectious diseases and the subsequent pathophysiology in the host environments, it has become extremely challenging to control infectious diseases, especially with the recent outbreaks of contagious infections [1, 2]. Addressing the AMR and the complicated infectious diseases associated with it entails multidimensional worldwide efforts to discover and develop new antimicrobials in a way to mitigate the resistance emergence.

Antipathogenic or antivirulence strategy is a new approach in this regard, in that it interferes with a virulence pathway that bacteria have exploited to cause diseases in the infected hosts [3, 4]. These are able to disarm the pathogens rather than to directly target growth or viability, unlike the conventional antibiotics. Due to this, antipathogenics or antivirulence compounds may have advantages, given that they limit collateral damage to the normal microflora and potentially engender a little or milder evolutionary pressure towards the resistance development. Some antipathogenic compounds have been discovered based on the novel targets from the growing knowledge about the molecular pathogenesis of bacteria, which include quorum-sensing and secretion systems [5, 6].

In Gram-negative bacteria, OxyR is the master peroxide sensor that regulates the transcription of the defence genes in response to H_2_O_2_. This transcriptional regulator is required for the full virulence of the opportunistic human pathogen, *Pseudomonas aeruginosa* [7, 8], being responsible for the optimal expression of phenazines and rhamnolipids as well as for the induced transcription of the antioxidant enzymes including the major catalase, KatA, critical for the acute virulence and intrinsic AMR of some *P. aeruginosa* strains [9–13]. We also elucidated the full-length structures of OxyR at reduced and oxidized states during its redox cycle, by showing all of the structural features describing the tetrameric assembly and an H_2_O_2_ molecule bound near the conserved cysteines [14]. These lines of works have led us to a proof-of-concept approach to use this virulence factor as a new antipathogenic target by identifying new molecules that impair the function of OxyR in responding to oxidative stress conditions.

In this study using the structural and functional features of the *P. aeruginosa* OxyR, we performed computer-based virtual screens to identify the chemical compounds that potentially bind to the OxyR regulatory domain, considering the predictive rules for penetration and accumulation in Gram-negative bacteria [15]. Among the hits whose treatment phenocopied the serial dilution defect of the *oxyR* null mutant, a compound was shown to affect the OxyR-induced transcription and its redox cycle involving disulfide bond formation. Most importantly, this compound specifically attenuated the virulence of *P. aeruginosa* in *Drosophila* systemic infections.

## Methods

### Data collection and preparation

The crystal structure of the *P. aeruginosa* OxyR from the Protein Data Bank (PDB code: 4Y0M) representing the reduced state of the regulatory domain (RD) spanning from 88th to 310th amino acids was selected as the target region and prepared for molecular docking studies by correcting the missing hydrogens and the unfilled valence atoms and then subjected to energy minimization by applying the CHARMm force field until a satisfactory gradient tolerance was obtained. For ligand preparation, 534 in-house compounds below an ALogP of 5 and molecular weight of 500 were prepared for virtual screening. The prepared ligands were converted to the MOL file format and their corresponding 3D structures were generated on the Discovery Studio 2019 (Accelrys Inc.). CHARMm force field was applied as a measure to minimize the ligand molecules.

### Molecular docking and virtual screening

Structure-based virtual screening by applying docking simulations was performed adopting the CDOCKER module of Discovery Studio 2019, which depends on CHARMm-based force field. The number of generated poses was set to 100 for each ligand, and default settings were selected for other parameters. The docking estimation was performed by the calculated CDOCKER energy, based on the internal ligand strain energy and receptor-ligand interaction energy. Additionally, CDOCKER interaction signifies the energy of the non-bonded interactions that exist between the ligand and the target region. Ten compounds were selected based on the value of CDOCKER interaction energy and the number of interactions (Table 1). The physicochemical parameters for permeability to Gram-negative bacterial cells and drug-likeliness properties were also calculated [15]: the globularity was calculated using the entryway website (http://www.entry-way.org) and the flexibility was predicted by the number of rotatable bonds calculated using Discovery Studio 2019. Drug-likeliness properties were evaluated by applying Lipinski’s rule of five [16].

**Table 1.** Virtual screening results showing the 10 selected compounds

Compound	CDOCKER energy*	CDOCKER interaction*	CHARMm energy*	No. of interactions	Globularity*†*	Flexibility*†*	ALogP‡	Molecular wt*‡*	No. of H bond acceptors*‡*	No. of H bond donors‡
1	8.87601	19.2097	7.26845	6	0.102	5	1.541	291.326	6	2
2	−209.866	−57.9865	−23.6281	11	0.065	8	1.133	366.392	9	4
3	7.79472	11.7873	3.47962	3	0.1	2	2.568	224.258	3	2
4	−71.5341	−33.5218	−3.45211	3	0.041	4	3.135	316.353	4	3
5	−292.27	−93.9394	−12.5693	5	0.05	6	3.183	346.379	3	2
6	−356.24	−20.734	−4.89623	7	0.088	5	1.47	336.366	8	3
7	−437.05	−94.5293	4.62023	10	0.101	5	2.387	376.43	8	3
8	−459.509	−114.789	−67.6858	10	0.047	9	1.526	408.429	9	3
9	11.3658	20.379	−7.62892	3	0.024	1	2.717	306.721	4	0
10	−704.499	−182.944	−8.8061	12	0.0677	4	3.808	322.401	4	3

*The energy values are expressed in unit of kcal/mol.

†The parameters associated with accumulation in Gram-negative bacteria.

‡The criteria of Lipinski’s rule of five parameters for drug-likeness evaluation.

### Compounds

All in-house compounds were synthesized using commercially available reagents and purified by flash column chromatography. The chemical structures were confirmed through proton NMR spectra and high-resolution mass spectrometry spectra.

### Bacterial strains and culture conditions

The bacterial strains used in this study are listed in Table S1 (available in the online version of this article). *P. aeruginosa* and *Escherichia coli* strains were grown at 37 °C using Luria-Bertani (LB) (1 % tryptone, 0.5 % yeast extract and 1 % NaCl) broth or on 2 % Bacto-agar solidified LB plates. Overnight-grown cultures were used as inoculum (1.6×10^7^ c.f.u. ml^−1^) into fresh medium and grown at 37 °C shaking incubator until logarithmic (OD_600_ of 0.7) growth phase, and then the cell cultures were used for the experiments described herein. For anaerobic growth, bacteria were grown in LB medium supplemented with 15 mM KNO_3_ in an anaerobic jar with AnaeroPack (MGC) [12].

### Phenotypic assay

A spotting assay was performed to investigate aerobic serial dilution as described previously [7]. Briefly, *P. aeruginosa* cells were grown to the logarithmic growth phase. Aliquots (3 µl) of cultures serially diluted by ten-fold in LB broth with or without chemical compounds (100 mM) were spotted onto LB agar plates. The plates were incubated at 37 °C for 18 h under aerobic conditions or for 32 h under anaerobic condition as described elsewhere [10].

### OxyR redox status assay

Thiol trapping by 4-acetamido-4′-maleimidylstilbene-2,2′-disulfonic acid (AMS) was used to investigate the changes in the OxyR redox status upon chemical treatment. OxyR proteins tagged with a FLAG epitope was used as described previously [11]. The *oxyR* null mutant cells containing FLAG-tagged OxyR were grown to logarithmic growth phase in LB broth with or without compound 1 (200 µM) at 37 °C. After 1 mM H_2_O_2_ treatment, an aliquot of the culture (1 ml) was mixed with 110 µl ice-cold 100 % (wt/vol) trichloroacetic acid (TCA) at various time points and harvested. The cell pellets were resuspended in 400 µl of 10 % TCA and disrupted by sonication. After centrifugation, the pellets were mixed with 40 µl of AMS buffer (0.5 M Tris-HCl [pH 8.0], 20 mM AMS, 2 % SDS, 100 mM NaCl, 1 mM EDTA, and 5 % glycerol) and incubated in the dark for 1 h prior to cell extract preparation, followed by Western blot analysis using anti-FLAG M2 antibody (Sigma).

### β-Galatosidase assay

pQF50-*katAp*, the pQF50-based *katA* promoter fusion containing both *katAp1* and *katAp2* promoters, was used in this study [10]. This construct was introduced by electroporation and β-galactosidase activity was determined using the cultures grown to mid-logarithmic (OD_600_ of 0.3) growth phase as previously elsewhere [11].

### Evaluation of antibacterial efficacy

*Drosophila* systemic infection was performed as previously described [17]. Briefly, *Drosophila melanogaster* strain Oregon R was grown and maintained at 25 °C using the corn meal-dextrose medium [0.93 % agar, 6.24 % dry yeast, 4.08 % corn meal, 8.62 % dextrose, 0.1 % methyl paraben, and 0.45 % (v/v) propionic acid]. For systemic infection, 4- to 5-day-old adult female flies were infected by pricking at the dorsal thorax with a 0.4 mm needle (Ernest F. Fullam, Inc.). The needle was dipped into PBS-diluted bacterial suspension containing either *P. aeruginosa* PA14 (10^7^ c.f.u. ml^−1^) or *Staphylococcus aureus* SA3 (10^8^ c.f.u. ml^−1^) grown to the OD_600_ of 3.0 in the presence or absence of compound 1 (200 µM). Survival rates of the infected flies were monitored for up to 48 h post-infection. Flies that died within 12 h were excluded in mortality determination. Mortality assay was repeated at least three times.

### Statistics

Statistical analysis was performed using GraphPad Prism version 6.0 (GraphPad Software, La Jolla, CA). Data for each analysis represents a set of three independent replicates. Statistical significance between the groups is indicated, based on a *P* value of less than 0.01 (*, *P* <0.01; **, *P* <0.005; ***, *P* <0.001) by using Kaplan-Meier log-rank test and Student’s *t*-test. Error bars represent the standard deviations.

## Results and Discussion

### Virtual screens for the chemical hits based on the OxyR structure

As an initial attempt to identify new antibacterial hits directly targeting the *P. aerguinosa* OxyR, a computer-based virtual screening was performed on the reduced form of the OxyR regulatory domain (RD). A total of 534 in-house chemical compounds were screened to identify ten hits based on the overall scores of receptor-ligand interactions such as CDOCKER interaction energy as well as the predicted physicochemical parameters for accumulation in Gram-negative bacteria (Table 1). It was reported as the ‘eNTRy rules’ that the lower globularity and flexibility (inversely proportional to number of rotatable bonds) and the charged primary amine functionality with localized polarity near the amine favour the penetration and accumulation inside the Gram-negative bacterial cells [15]. All ten compounds vary in the globularity and flexibility, but no compounds have charged primary amines.

Lipinski’s rule of five (RO5) was applied to evaluate the drug-likeness, i.e. the pharmacological properties as an orally active drugs in humans that are relatively small and moderately hydrophobic in general [16]. All the ten compounds satisfied the RO5, which include four criteria (Table 1): 1) molecular weight of less than 500 daltons, 2) number of hydrogen bond donors of less than 5, 3) number of hydrogen bond acceptors of less than 10, 4) calculated water partition coefficient (ALogP) of less than 5. The information might be useful for further validation to lower the attrition rates during the drug development process.

### *P. aeruginosa* displays serial dilution defects by compounds 1, 4 and 6

**Fig. 1.**
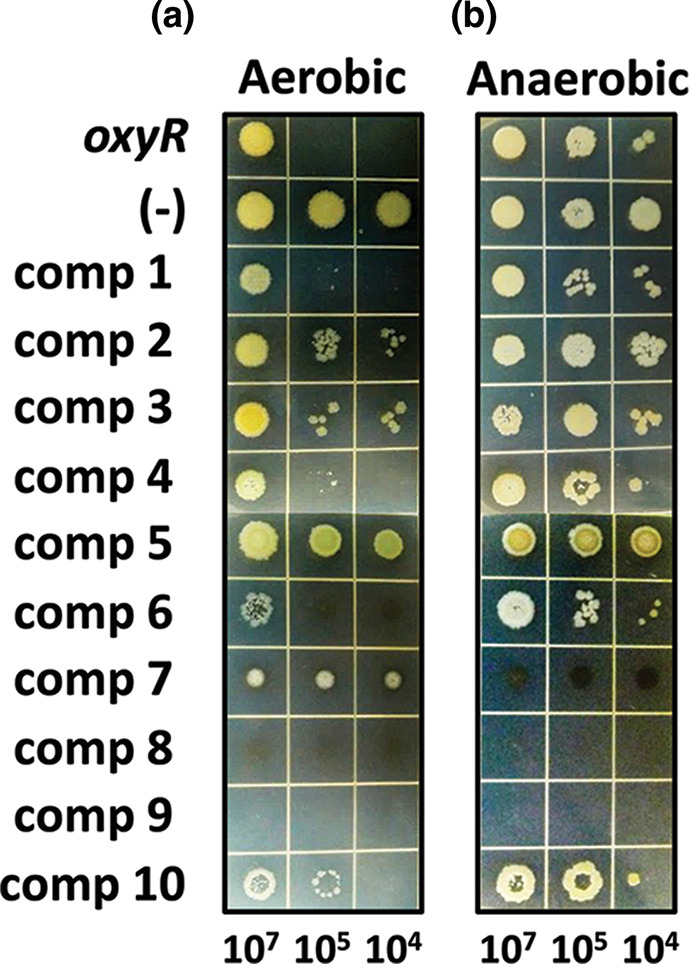
Experimental validation of the selected compounds. The wild-type (WT) PA14 cells were tested for the growth defect upon serial dilution in the presence of the 10 selected compounds in Table 1, which were performed under aerobic (**a**) and anaerobic (**b**) conditions. The *oxyR* null mutant (*oxyR*) was included as the control. The numbers at the bottom indicate the colony-forming units (c.f.u.s) of the cell spots.

Next, we have experimentally tested the 10 selected hits for their activity to modulate the OxyR functions. The *oxyR* null and some point mutants display defective growth only by serial dilution under aerobic condition [7]. Three out of the 10 compounds (1, 4, and 6) affected the wild-type cell growth similarly to the *oxyR* null mutant, whereas the cells were affected to a lesser extent by compounds 2, 3, and 10 (Fig. 1). The growth of the wild-type and the *oxyR* mutant bacteria were not affected at all by these six compounds, whereas three compounds (7, 8, and 9) were growth-inhibitory. Compound 5 had neither affected the growth nor caused the aerobic serial dilution defect of the wild-type bacteria.

**Fig. 2.**
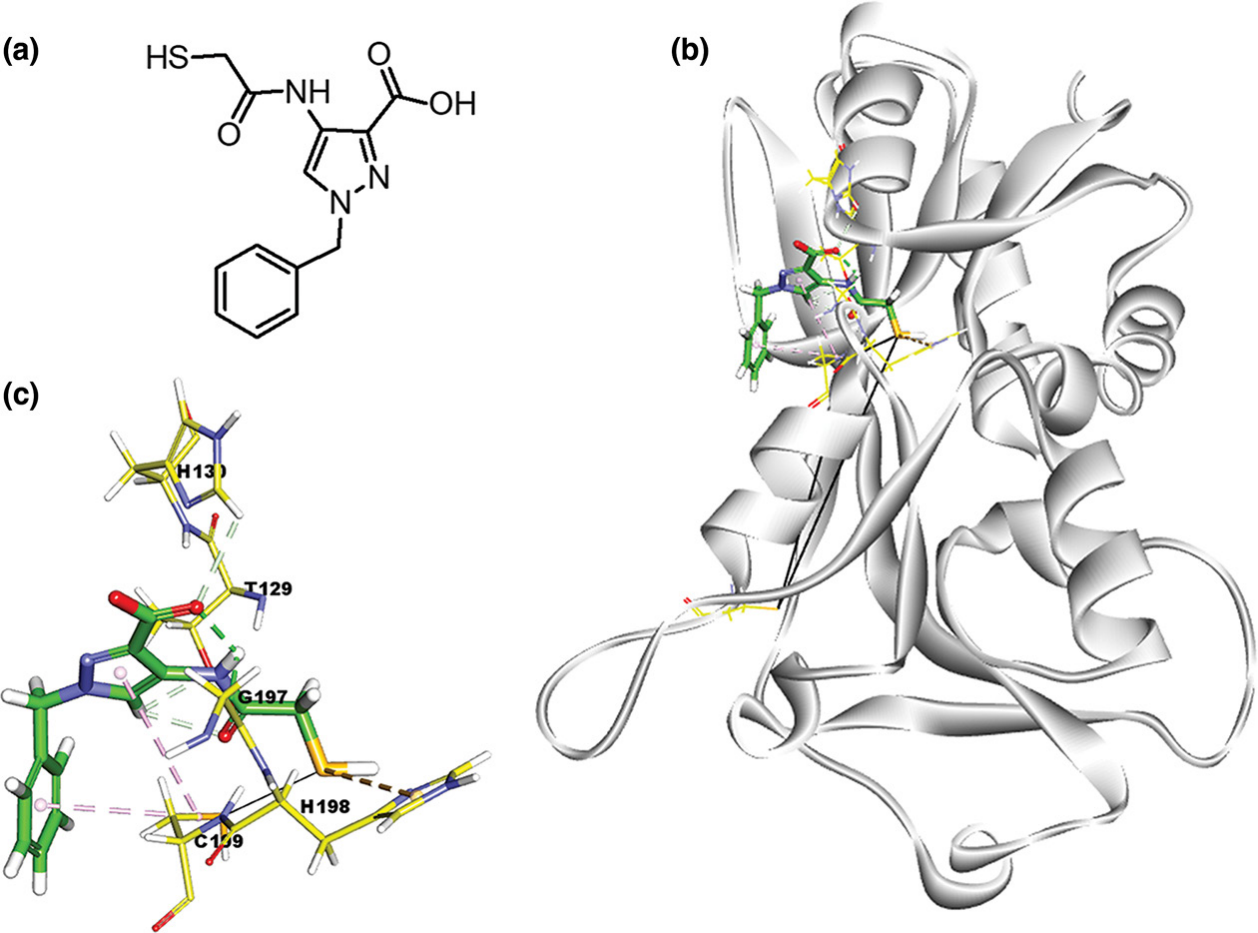
Structural modelling of the interactions between compound 1 and OxyR . (**a**) Chemical structure of compound 1. (**b**) Molecular docking between compound 1 (coloured) and the reduced form of OxyR RD (light grey). (**c**) Molecular interactions within the binding pocket of OxyR and interacting amino acid residues (Thr129-His130 and Gly197-His198-Cys199) in one-letter code. The bonding distance cutoff is 5.0 Å. The green dotted line indicates the hydrogen bond, the pink dotted lines indicate the pi-alkyl bond involving Cys199, and the brown dotted line indicates the pi-sulphur interaction involving the thiol moiety of compound 1.

From the predicted parameters shown in Table 1, the highest CHARMm-energy compound (one) out of the three hits was selected for further analysis, whose treatment phenocopied the *oxyR* null mutant. It is noteworthy that this compound possesses free thiol moiety (Fig. 2a), which might be able to react with the cysteine residues of the OxyR RD. The molecular docking analyses of compound 1 by using crystal structure of the OxyR revealed the relatively stable interactions between compound 1 and the OxyR RD. In the best-docked pose as shown in Fig. 2b, compound 1 engaged in a total of six interactions with OxyR (Table 1, Fig. 2c): it participated in three hydrogen-bonding interactions with Thr129, His130, and Gly197 around the pyrazole centre. In particular, the thiol moiety was deeply projected toward the H_2_O_2_-binding region and formed pi-sulphur interaction with His198, which is noteworthy in that His198 is critical for the reactivity of the peroxidatic cysteine (Cys199) [14]. It should be noted as well that the pyrazole and the benzyl groups participated in two pi-alkyl interactions with the thiol group of Cys199. Relatively tight packing between compound 1 and the surrounding amino acid residues made substantial contribution to the overall stability of the interactions between compound 1 and OxyR. These results suggest that compound 1 could bind to the bipartite regions (Thr129-His130 and Gly197-His198-Cys199) at the OxyR RD, which might compromise the function of OxyR in a way to phenocopy the *oxyR* null mutant phenotypes rather than those of the *oxyR* RD point mutants [7].

### OxyR-mediated transcriptional regulation is impaired by compound 1

**Fig. 3.**
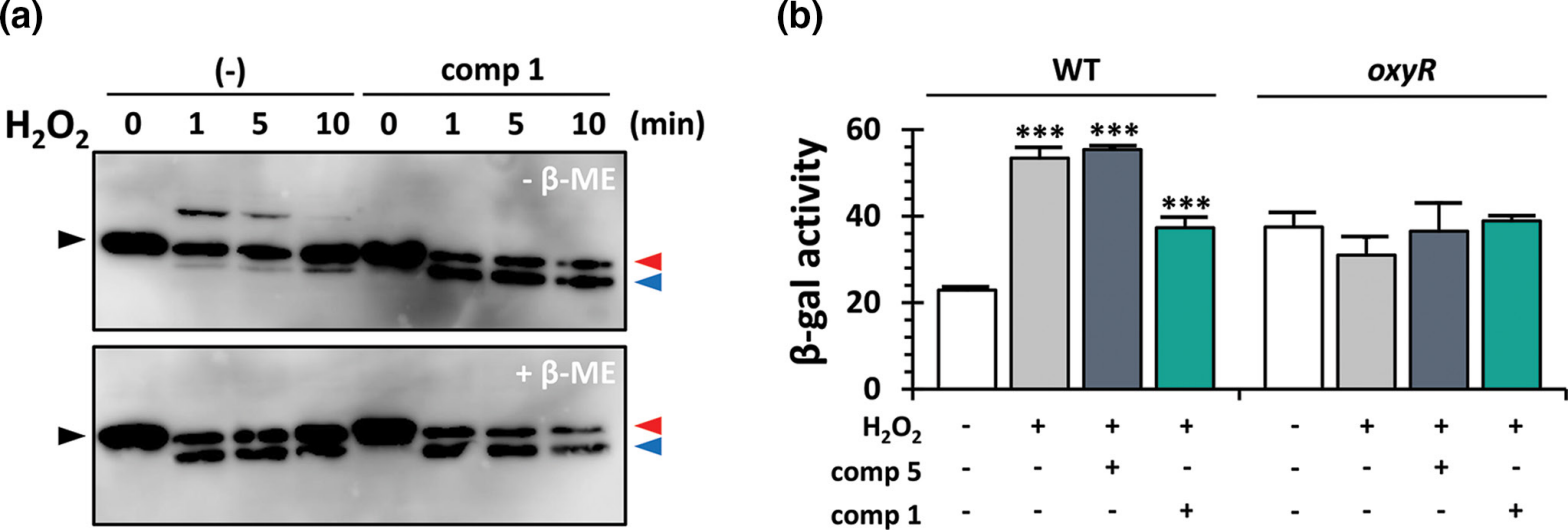
Redox cycle of OxyR in the presence of compound 1. (**a**) Effect of compound 1 on the redox cycle of OxyR. The redox status of OxyR was monitored using the cells harbouring FLAG-tagged OxyR that had been grown in the presence or absence of compound 1 (200 µM). Cells were harvested before (0 min) or after (1, 5, and 10 min) treatment with 1 mM H_2_O_2_ followed by alkylation with 20 mM AMS as described elsewhere [11] The OxyR redox states were observed under non-reducing (i.e. without 2-mercaptoenthanol (β-ME); − β-ME) or reducing (i.e. with β-ME; + β-ME) conditions. The faster migrating bands upon compound 1 treatment have been designated by red and blue arrow heads in comparison with the reduced-state band (black arrow head). (**b**) Effect of compound 1 on the *katA* transcription. The *katA* transcription was monitored by β-galactosidase activity for the wild-type (WT) and the *oxyR* mutant bacteria harbouring the pQF50-derived *lacZ* transcriptional fusion with the dual *katA* promoters [10]. Cells were grown to mid-logarithmic growth phase with (+) or without (-) compound 1 or 5 and then treated with (+) or without (-) H_2_O_2_ for 20 min. The β-galactosidase activities are measured and expressed as Miller units, with standard deviations from three independent experiments. The statistical significance based on Student’s *t*-test is indicated (***, *P* <0.001).

Since compound 1 is supposed to bind to the OxyR RD (Fig. 2b, c) and presumably compromise the OxyR function (Fig. 1), we investigated the potential impact of compound 1 on the redox cycle of OxyR under peroxide stress conditions *in vivo*. We previously observed that OxyR undergoes three distinct oxidized states, as assessed by thiol alkylation with AMS for the OxyR proteins tagged with a FLAG epitope at their C terminus [11]: two faster migrating species I and II than the reduced OxyR and a ~50 kDa species (III). As shown in Fig. 3a, three oxidized OxyR species were clearly observed under non-reducing conditions without 2-mercaptoenthanol (β-ME) treatment and most of the oxidized species were rapidly reduced *in vivo* within 10 min after H_2_O_2_ exposure. Since two prominent bands were observed under reducing condition (i.e. with β-ME treatment), it is probable that the species I might possess three free thiols and the species II and III might possess two free thiols, both of which migrate faster than the reduced species with four free thiols. Although the detailed characterization of the oxidized species requires more extensive chemical identification based on mass spectrometry [18], it is evident that more than two types of disulfide bonds are formed during the OxyR peroxide sensing.

In contrast, however, the oxidized states of OxyR proteins in the presence of compound 1 appeared to significantly differ at least in three aspects: first, the species III was not observed at all; second, more fastest migrating band was observed; lastly, no rapid reduction was observed within 10 min. Most importantly, there is no apparent difference in the band profiles between non-reducing and reducing conditions, which suggest that the disulfide bonds were not formed in the presence of compound 1 during the H_2_O_2_ treatment. These results and the proposed direct interaction between compound 1 and the OxyR RD led us to the tentative conclusion that the compound 1 actually binds to OxyR and that the ligand binding hamper the disulfide bond formation involving the OxyR RD, which may be able to compromise the OxyR function in an unknown mechanism.

We have also confirmed the effect of the compound 1 on the OxyR function by using the *katA-lacZ* fusion as described in Methods, in that OxyR acts as both an activator and a repressor for the *katA* gene [11]. As shown in Fig. 3b, the overall transcription from the dual *katA* promoters were induced in response to H_2_O_2_ treatment in the absence of compound 1 or in the presence of the control chemical (compound five). However, the transcription level was partially reduced by compound 1 to the level observed for the *oxyR* mutant, which is in agreement with the observation that compound 1 treatment resulted in the *oxyR* null mutant phenotype (Fig. 1). It has been verified that the point mutants for peroxidatic and resolving cysteines act like either constitutive activator (for Cys199 mutants) or unresponsive (i.e. locked) repressor (for Cys208 mutants) [11]. It should be noted that compound 1 was able to deplete the OxyR function, although it targets the OxyR RD in a way to affect the redox cycle of OxyR (Figs. 1 and 3). This could be further elucidated with more precise understanding of the complicated molecular details in the redox cycle of OxyR during peroxide sensing which need to be systemically addressed in regards to Cys modifications as well as the functions of the amino acid residues (Thr129, His130, Gly197, His198, and Cys199) that are supposed to interact with compound 1.

### Compound 1 displays antibacterial efficacy against *P. aeruginosa*

**Fig. 4.**
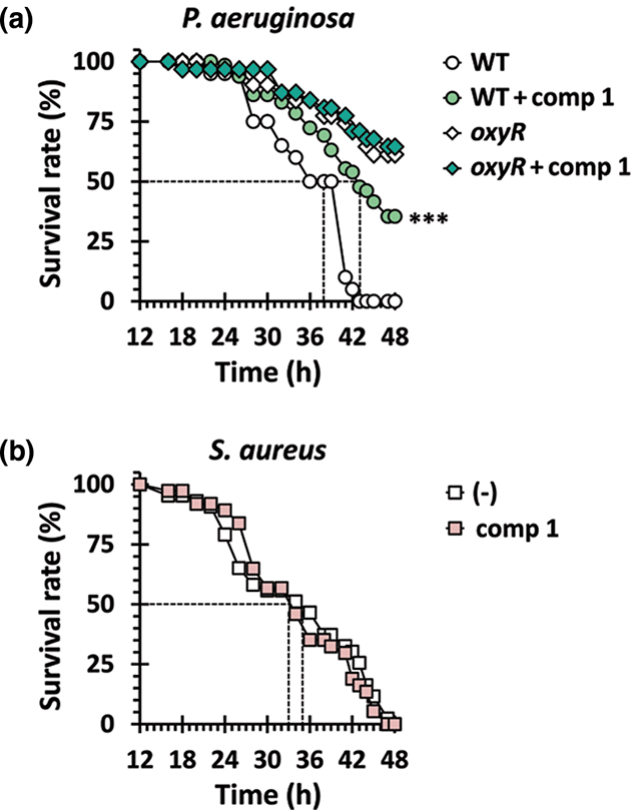
Antibacterial efficacy of compound 1 against bacterial pathogens. Mortality of infected flies upon administration of compound 1 were measured. The Oregon R flies were systemically infected with *P. aeruginosa* wild-type (WT) and the *oxyR* mutant (**a**) and *S. aureus* SA3 (**b**) cells that had been pre-treated with nothing (empty) or 200 µM of compound 1 (filled). The infected flies were transferred to the new media and the survival rates were determined over time. The dotted lines represent the time required to reach 50 % mortality. The statistical significance based on a log-rank test is indicated (***, *P* <0.001).

Since OxyR is required for the full virulence of *P. aeruginosa* [7, 8], it is possible that compound 1 could affect the virulence traits of *P. aeruginosa *. To test for this possibility, we used the *Drosophila* systemic infection model to evaluate the antibacterial efficacy of compound 1, as exploited in our previous studies [7, 10]. Considering the solubility of the compound in fly media, pre-treated bacterial cells with compound 1 were injected by pricking as described in Methods. As shown in Fig. 4a, the virulence attenuation was clearly observed by compound 1 treatment for the *P. aeruginosa *-infected flies in our experimental condition. It should be noted that the attenuated virulence of the *oxyR* mutant was not further affected at all, corroborating the effect of compound 1 might be OxyR-dependent.

We also noted that the compound 1 treatment did not display antibacterial efficacy against the methicillin-resistant *S. aureus *, SA3 [19] (Fig. 4b). SA3 or other Gram-positive bacterial pathogens possess PerR, a Fur paralog, as the peroxide sensor, whereas OxyR is conserved among the Gram-negative bacterial species [7, 20]. Since we had failed to identify the antibacterial efficacy against SA3 in any experimental infection conditions we had tested, it should be concluded that compound 1 specifically impaired the OxyR function and thus specifically target the Gram-negative bacterial pathogens that possess OxyR as the master peroxide sensor.

### Conclusions

The idea of inhibiting virulence pathways by exploiting small chemical compounds has been recently conceptualized as an effective means to control bacterial infections, in regards to reducing AMR emergence. Antipathogenic or antivirulence compounds that have been developed so far include the small molecules that target adherence, toxins and quorum sensing [20–22]. Those targets are present at the outside of the bacterial cells in common, which might be due to the advantage of those extracellular targets in circumventing the permeability issues of the chemical hits for better druggability. The growing knowledge about both the bacterial virulence factors from basic pathogenesis research and the druggability information from pharmacology and pharmaceutics research enables us to expand our attention to the new horizon of the vulnerable intracellular targets for antipathogenic therapy.

In this study based on the functional and structural information of the intracellular peroxide sensor, OxyR, we have demonstrated the proof-of-concept of using OxyR as the antipathogenic target by identifying a chemical inhibitor. The recent understanding of the predictive rules (i.e. the eNTRy rules) for penetration and accumulation of small molecules in Gram-negative bacteria was harnessed as well for virtual screening by considering the globularity and the flexibility of the chemical compounds [15]. This might account for the relatively high hit rates (i.e. 3 or 6 out of 534 compounds) observed in the present study. Virtual screening was verified by *in vitro* experiments that is relatively simple for OxyR, in that the *oxyR* null mutant of *P. aeruginosa* showed various discernable *in vitro* phenotypes, most notably, the aerobic serial dilution defect. Molecular docking data of one hit were also supported in part by the redox status assay, although further biochemical studies are needed to delve into the mechanistic aspects of the chemical-protein interactions. We still have more hits to elaborate on for further characterization. This will be further geared by reverse genetic approaches in collaboration with medicinal chemistry ones. Based on these, the identified hits can be further optimized for a first-in-class antibacterial drug candidate, which disarm the oxidative stress response networks during the infections caused by Gram-negative bacteria. More importantly, the whole procedure described in the present study will help identify new chemical hits effectively targeting the intracellular virulence factors in multi-drug resistant Gram-negative bacterial pathogens.

## Supplementary Data

Supplementary material 1
