# Supplementary material for: An antipathogenic compound that targets the OxyR peroxide sensor in Pseudomonas aeruginosa
Source: J Med Microbiol. 2021 Apr 8;70(4):001341. doi: 10.1099/jmm.0.001341 (PMC8289212; doi:10.1099/jmm.0.001341)
Supplement: Supplementary material 1 [file jmm-70-1341-s001.pdf]

An antipathogenic compound that targets the OxyR peroxide sensor in *Pseudomonas aeruginosa*

Hyo-Young Oh,[1†] Shivakumar S. Jalde,[2†] In-Young Chung,[1†] Yeon-Ji Yoo,[1] Hye-Jeong Jang,[1] Hyun-Kyung Choi,[2*] and You-Hee Cho[1*]

[1]Department of Pharmacy, College of Pharmacy and Institute of Pharmaceutical Sciences, CHA University, Gyeonggi-do 13488, Korea and [2]Department of Medicinal Chemistry, Jungwon University, Goesan 28024, Korea

[†]H.-Y. Oh, S.S. Jalde, and I.-Y. Chung contributed equally to this work.

[*] To whom correspondence should be addressed. Tel: +82 31 881 7165; Fax: +82 31 881 7219; E-mail: youhee@cha.ac.kr
Correspondence may also be addressed to Hyun-Kyung Choi. Tel: +82 43 830 8682; Fax: +82 43 830 8682; E-mail: hkchoi@jwu.ac.kr

Running Title: An antipathogenic hit targeting OxyR
Keywords: *Pseudomonas aeruginosa*, OxyR, antipathogenic, oxidative stress, virulence

Table S1. Bacterial strains and plasmids used in this study

Strain or plasmid	*Relevant characteristics or purpose*[a]	*Reference or source*
Pseudomonas aeruginosa		
PA14	wild type laboratory strain; Rif^R	Lab collection
oxyR	PA14 with in-frame deletion of *oxyR*; Rif^R	Choi et al. 2007
Staphylococcus aureus		
SA3	Methicillin-resistant laboratory strain; Mc^R	Jang et al. 2019
Escherichia coli		
DH5α	multi-purpose cloning	Lab collection
Plasmids		
pUCP18	general purpose cloning in *P. aeruginosa*; Cb^R	Lab collection
pUCP18-oxyR-FLAG	pUCP18 with the C-terminally FLAG-tagged OxyR; Cb^R	Heo et al. 2010
pQF50	*lacZ* transcriptional fusion; Cb^R	Lab collection
pQF50-*katAp*	pQF50 with the dual *katA* promoters; Cb^R	Chung et al. 2016

[a] Rif^R, rifampicin-resistant; Mc^R, methicillin-resistant; Cb^R, carbenicillin- and ampicillin-resistant.

REFERENCE

Choi Y-S, Shin D-H, Chung I-Y, Kim, S-H, Heo Y-J et al. Identification of *Pseudomonas aeruginosa* genes crucial for hydrogen peroxide resistance. *J Microbiol Biotechnol* 2007;17:1344-1352.

Chung I-Y, Kim B-o, Jang H-J, Cho Y-H. Dual promoters of the major catalase (KatA) govern distinct survival strategies of *Pseudomonas aeruginosa*. *Sci Rep* 2016;6:31185.

Heo Y-J, Chung I-Y, Cho W-J, Lee B-Y, Kim J-H et al. The major catalase gene (KatA) of *Pseudomonas aeruginosa* PA14 is under both positive and negative control of the global transactivator OxyR in response to hydrogen peroxide. *J Bacteriol* 2010;192:381-390.

Jang H-J, Chung I-Y, Lim C, Chung S, Kim B-o et al. Redirecting an anticancer to an antibacterial hit against methicillin-resistant *Staphylococcus aureus*. *Front Microbiol* 2019;10:350.
